# Suppression of *E. multilocularis* Hydatid Cysts after Ionizing Radiation Exposure

**DOI:** 10.1371/journal.pntd.0002518

**Published:** 2013-10-24

**Authors:** Xin Zhou, Yumin Zhao, Rong Zhou, Hong Zhang

**Affiliations:** 1 Department of Heavy Ion Radiation Medicine, Institute of Modern Physics, Chinese Academy of Sciences, Lanzhou, China; 2 Key Laboratory of Heavy Ion Radiation Biology and Medicine of Chinese Academy of Sciences, Lanzhou, China; 3 Key Laboratory of Heavy Ion Radiation Medicine of Gansu Province, Lanzhou, China; 4 Department of Parasitology, Guilin Medical University, Guilin, Guangxi, China; University at Buffalo, The State University of New York, United States of America

## Abstract

**Background:**

Heavy-ion therapy has an advantage over conventional radiotherapy due to its superb biological effectiveness and dose conformity in cancer therapy. It could be a potential alternate approach for hydatid cyst treatment. However, there is no information currently available on the cellular and molecular basis for heavy-ion irradiation induced cell death in cystic echinococcosis.

**Methododology/Principal Findings:**

LD50 was scored by protoscolex death. Cellular and ultrastructural changes within the parasite were studied by light and electron microscopy, mitochondrial DNA (mtDNA) damage and copy number were measured by QPCR, and apoptosis was determined by caspase 3 expression and caspase 3 activity. Ionizing radiation induced sparse cytoplasm, disorganized and clumped organelles, large vacuoles and devoid of villi. The initial mtDNA damage caused by ionizing radiation increased in a dose-dependent manner. The kinetic of DNA repair was slower after carbon-ion radiation than that after X-rays radiation. High dose carbon-ion radiation caused irreversible mtDNA degradation. Cysts apoptosis was pronounced after radiation. Carbon-ion radiation was more effective to suppress hydatid cysts than X-rays.

**Conclusions:**

These studies provide a framework to the evaluation of attenuation effect of heavy-ion radiation on cystic echinococcosis in vitro. Carbon-ion radiation is more effective to suppress *E. multilocularis* than X-rays.

## Author Summary

Surgical removal of cysts may be impractical in cases that cysts are in multiple organs or tissues, or in risky locations. In that case, alternative treatment should be employed. Heavy-ion radiation could be an effective way for treatment of hydatid cysts, taking its full advantage of well-defined range, small lateral beam spread and an enhanced biological effectiveness. In this study, we found that carbon-ion radiation could result in extensive mitochondrial DNA damage and apoptosis in hydatid cysts. Cellular and ultrastructural changes were observed after ionizing radiation, which were indicative of cysts growth inhibition. To our knowledge, this is the first study reporting the biological effect of carbon-ion radiation on *E. multilocularis* hydatid cysts.

## Introduction

Alveolar echinococcosis, which is caused by larvae of *E. multilocularis*, is common in Europe, China, and Siberia. It's a serious disease to human that has a significantly high fatality rate. The most common form of treatment for cystic echinococcosis is surgical removal of the cysts combined with chemotherapy using albendazole and/or mebendazole. However, it would not be practical for surgery if cysts are in risky locations or in multiple organs or tissues. In this case, PAIR (puncture-aspiration-injection-reaspiration) combined with chemotherapy become an alternative options of treatment [1]. There are currently new studies looking for new treatment by mean of high-energy electromagnetic waves (such as X-rays). *In vivo* and *in vitro* studies shows that X-rays radiation could significantly increase protoscolex mortality and inhibit cysts growth [2], [3]. Recent case study represented radiation therapy could be an alternative treatment modality for hydatid disease of the chest wall after medical and surgical therapy failure [4]. However, conventional megavolt photon therapy such as X-rays may not be a good choice for hydatid cyst treatment, because *E. multilocularis* is mostly localized in the liver and lungs and the therapeutic effect of X-rays is limited and unsatisfactory on liver and lungs on the basis of their poor tolerance to irradiation [5]–[7].

Heavy-ion radiotherapy has been proved to be an ideal treatment for lung cancer and hepatocellular carcinoma due to its excellent local control and survival in cancer patients [8], [9]. Higher-energy beams of charged nuclear particles could offer significant advantages for the deep-seated hydatid cysts treatment in comparison to conventional megavolt photon therapy. Heavy-ion radiation could be an effective way for treatment of hydatid cysts, taking its full advantage of well-defined range, small lateral beam spread and an enhanced biological effectiveness.

Until now there are no *in vitro* studies reported the cellular and molecular effect of heavy-ion radiation on hydatid cysts. This is the first report on the cellular damage induced by carbon-ion irradiation in hydatid cysts.

## Materials and Methods

### Ethics statement

All animals experiments described have been conducted according to the Guideline on the Humane Treatment of Laboratory Animals stipulated by the Ministry of Science and Technology of the People's Republic of China (MOST) and were approved by the Institutional Animal Care and Use Committee (IACUC) of Institute of Modern Physics, CAS for the use of laboratory animals. The Regulations for the Administration of Affairs Concerning Experimental Animals (1988.11.1) is affiliated with Institute of Modern Physics, CAS.

### Sample collection

Hydatid cysts of *E. multilocularis* were freshly isolated from livers of female mice after secondary infection with hydatid cyst tissue homogenates, which were originally obtained from a sheep naturally infected with *E. multilocularis* in Qinghai, China. Collected cysts were rinsed with sterile saline for several times and then cultivated with RMPI 1640 medium at 37°C in a 5% CO_2_ incubator.

### Radiation procedure

X-rays were generated with an X-rays machine (FAXITRON RX650, USA) operated at 130 keV. An exposure-rate meter (AE-1321 M, Applied Engineering Inc, Japan) was used for the dosimetry. The dose rates were 1.3 Gy/min. Carbon-ion irradiations were performed at room temperature at the Heavy Ion Accelerator Center (HIRFL) of the Institute of Modern Physics (Lanzhou China) with 300 MeV/n carbon ions; the LET value for carbon ions was 40 KeV/µm. The dose rates were 1 Gy/min.

### LD50 assay

LD50 for hydatid cysts was monitored by protoscolex death after irradiation. The viability of protoscoleces 24 h after irradiation was assessed by observation under microscopy. The corresponding numbers of viable/non-viable protoscoleces were determined in 10 randomly chosen fields by phase contrast microscopy at 10×magnification.

### Light and electron microscopy

Cysts were fixed in 4% glutaraldehyde (24 h) and 1% osmium tetroxide sequentially, dehydrated with acetone gradient, and embedded in Epon 812 epoxy resin. The 60-nmsections were cut with an ultrathin section machine, stained with uranium and lead electron stains, and observed under the TEM. Cysts were also observed under light microscopy according to standard protocol.

### Immunoblotting

Western blotting protocol followed standard protocol. Briefly, protoscoleces were lysed in buffer containing 50 mmol/l Tris at pH 7.4, 50 mmol/l NaCl, 0.1% Triton X-100, 0.1% SDS, 0.3 mmol/l sodium orthovanadate, 1 mmol/l dithiotheritol, 10 mg/l leupeptin, and 5 mg/l aprotinin. Protein concentrations of lysates were determined using a BCA protein assay kit (Pierce, Rockford, IL, USA). An aliquot of each extract (40 µg protein) was fractionated by electrophoresis in an SDS–polyacrylamide gel and transferred to a PVDF membrane. Membranes were blocked with 10 ml TBST containing 0.5 g FBS at room temperature for 2 h, followed by incubation with antibodies against Caspase-3 (Biosynthesis Biotechnology, Beijing, China) at 4°C overnight. After washing with TBST for 30 min, appropriate HRP-conjugated secondary antibody was added to the membranes, which were incubated at room temperature for 1 h. Membranes were washed three times for 15 min each with TBST. Reactive proteins were visualized using a chemiluminescence kit (Santa Cruz Biotechnology, Santa Cruz, CA) according to the manufacturer's instructions.

### Caspase-3 activity assay

Detections were performed according to a previous description using the caspase-3 activity colorimetric assay kit instruction (Beyotime Institute of Biotechnology, Jiangsu, China) [10]. Briefly, 3-mg samples were added with 100-µl lysis buffer, ground, kept on ice for 15–20 min and centrifuged at 4°C, 17,000×g for 15 min. The supernatants were harvested and added into the reaction system on an assay plate with a control group according to the kit's instruction; plates were incubated at 37°C for 15 h and detected with a microplate reader for the absorbance at 405 nm (A405). The activated caspase-3 in samples catalyzed colorless substrate Ac-DEVD-pNA into yellow *p*NA: which concentrations could then be calculated according to *p*NA standard curve and sample A405; the activity of caspase-3 in samples was finally deduced based on the *p*NA concentration.

### MtDNA damage assay

Long PCR for mtDNA damage evaluation was performed using the GeneAmp XL PCR kit (PerKin–Elmer, Boston, MA). Quantitative long PCR were performed in an Eppendorf Mastercycler PCR system (Eppendorf, Hamburg, Germany). The PCR cycle test was performed before to ensure the PCR in the exponential phase. The sequence information of the primers was listed in Table 1. Briefly, a highly conserved nuclear single copy nuclear gene, DNA-directed RNA polymerase II (rpb2), was used as a reference for nuclear DNA copy number; Cytochrome c oxidase subunit II (Cox2), which encode an essential part of mitochondrial Electron Transport Chain (ETC), was used for mtDNA copy number reference. The PCR was initiated with a 75°C hot-start addition of the polymerase and allowed to undergo the following profile: an initial denaturation for 1 min at 94°C followed by 25 cycles for large fragments or 20 cycles for small fragments of 94°C denaturation for 15 sec and 68°C extension for 15 min. A final extension at 68°C was performed for 10 min at the completion of the profile. An aliquot of each PCR product was resolved on a 1% vertical agarose gel and electrophoresed in TBE for 4 hr. The gels were then digitally photographed and quantified with FluorChem FC2 (Alpha Innotech corporation). The DNA damage was quantified by comparing the relative level of amplification of the large fragments of mtDNA (8761 bp) normalizing this to the amplification of smaller (126 bp) fragments.

**Table 1:** Primers information.

	Primer sequences	Product size (bp)
mtDNA large frament	F: TTAGGCACATCAAACCGTAGR: AACCAAACCTCTCCACGAG	8761
cox2	F: TGCTGTTCCTGGTCGTCTTR: CCAACAACCTCCACAACAATAG	126
rpb2	F: TGAGGAGTGGAGTATGGAGAACR: AGCAGCCGTTGACGAAGAT	123

### Statistical analysis

Statistical analysis was performed on the means of the data obtained from at least three independent experiments. Data are presented as means±SD. Student's t-test program in Microsoft Excel was used to detect statistical significance. p<0.01 was considered to be statistically significant.

## Results

### Effect of ionizing radiation on cydatid cysts survival

**Figure 1:**
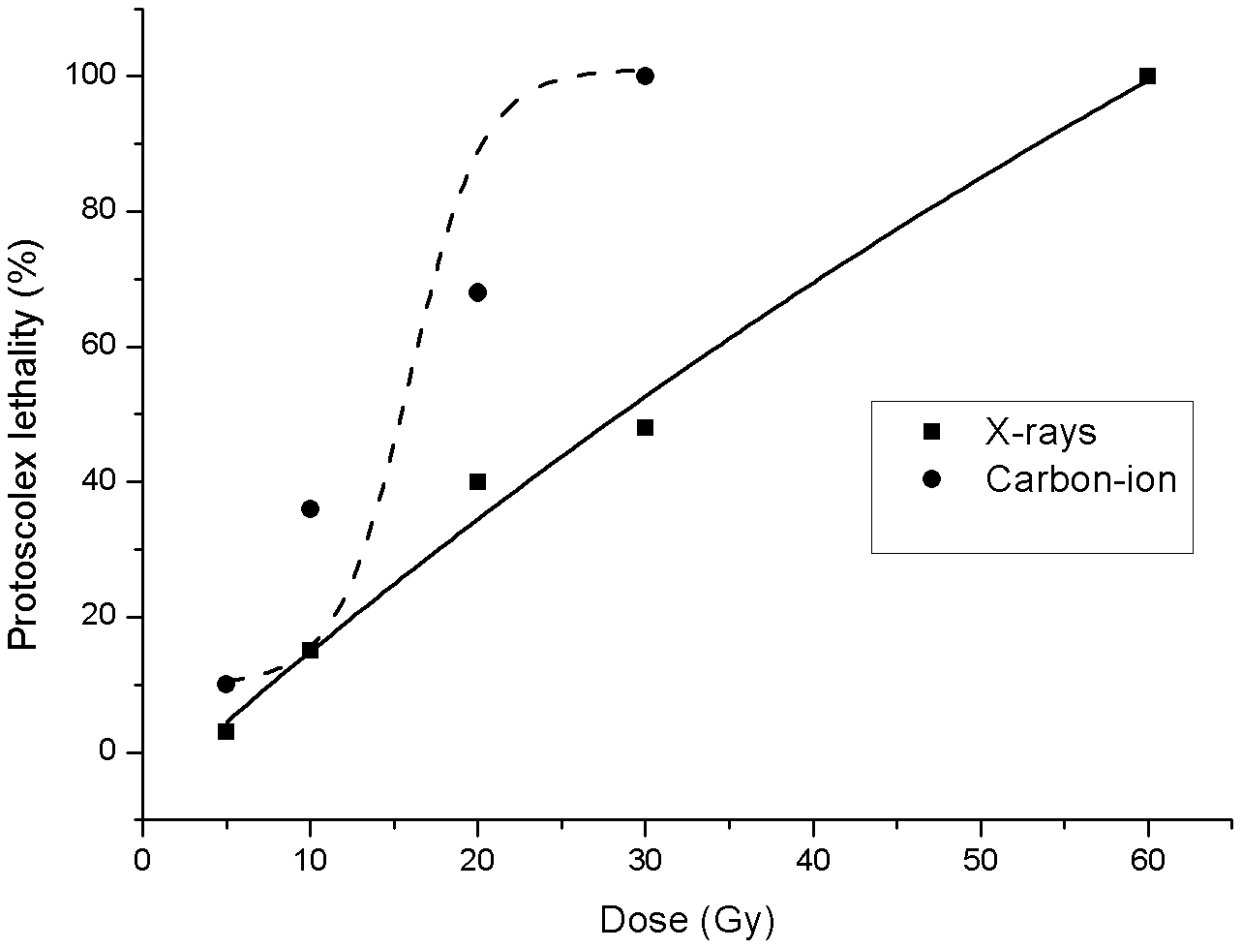
Percent lethality versus radiation dose. The data are fitted to a sigmoidal dose response function: *y = a+b/(1+exp(-(x(-c)/d))*, where *y* is the percent lethality, *x* is the dose in Gy, respectively.

The decrease in the vitality of the protoscoleces became evident after 3 h following the radiation exposure, since their movements has deceased (data not shown). Loss of protoscolex viability in IR-treated cultures became significant after 24 h, with a 100% protoscolex death at 60 Gy X-rays radiation and at 30 Gy carbon-ion radiation, respectively. The dose lethality after ionizing radiation is presented in Figure 1. The LD50 was 28.5 Gy for X-rays and 15.5 Gy for carbon-ion, respectively. The LD50 of X-rays irradiated cysts was significantly higher than that for carbon-ion irradiated cysts.

### MtDNA damage repair and mtDNA copy number in irradiated hydatid cysts

**Figure 2:**
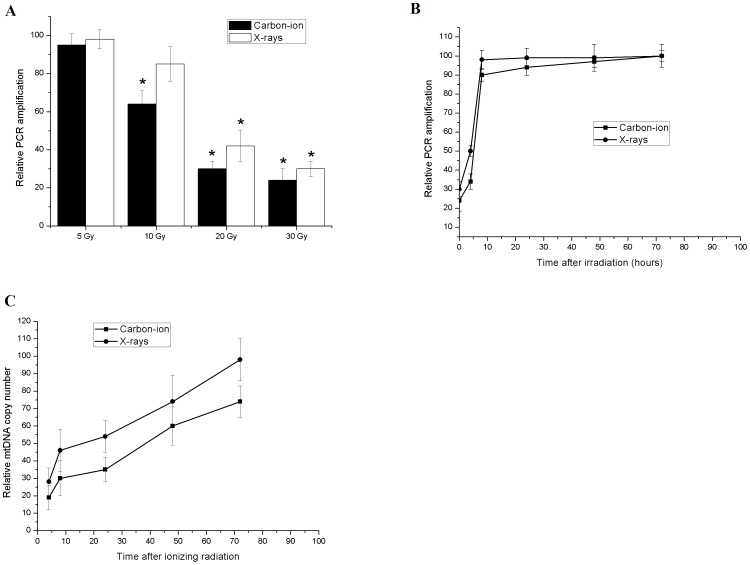
DNA damage repair and mtDNA copy number in irradiated hydatid cysts. (A) Quantification of mtDNA damage by long-PCR amplification of total DNA isolated from *E. multilocularis* hydatid cysts after 5–30 Gy X-rays or carbon-ion radiation. MtDNA damage was indicated by reduced PCR amplification. (B) Repair kinetics of mtDNA in *E. multilocularis* hydatid cysts within 72 hours after 30 Gy X-rays or carbon-ion radiation. (C) Quantification of mtDNA copy number by real-time PCR. Error bars represent the SD, each done in at least triplicate. *Statistical significant at p<0.01.

Both X-rays and carbon-ion radiation induced dose-dependent mtDNA damage. Carbon-ion radiation caused relative higher mtDNA damage than X-rays at the same dose (Figure 2A). MtDNA damage was repaired equal efficiently in both groups (Figure 2B).

Although mtDNA damage was completely repaired within 8 hours after radiation, we noticed significant decrease in the reference signal of cox2 4 hours after radiation, which was indicative of mtDNA copy number reduction. MtDNA copy number was then quantified by real-time PCR. Results showed that mtDNA copy number was dramatically reduced following radiation (up to 30%) and persisted for 24–48 hours before their gradual recovery (Figure 2C).

### Morphological alterations of irradiated parasites

**Figure 3:**
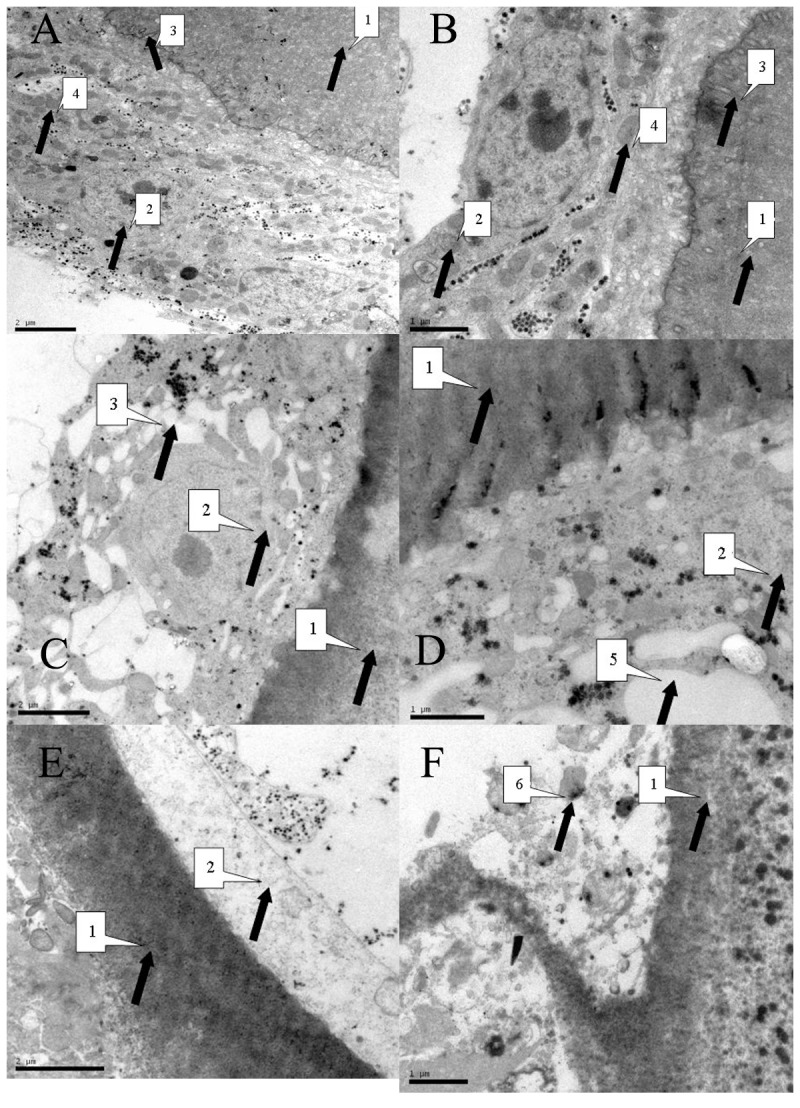
Representative thin-section electron microscopy of *E. multilocularis* hydatid cysts from control and irradiated cultures. Examples of control (A and B), 30 Gy X-rays irradiated (C and D) and 30 Gy carbon-ion irradiated (E and F) parasites are shown. A and B, the majority of the parasites displayed organized internal structures and their organelles were intact and clear. C—F, in irradiated samples, parasites displayed abnormal morphologies that included clumped organelles, abnormally large vacuoles, and generally disorganized structure. 1: germinal layer; 2: laminated layer; 3: border between germinal layer and laminated layer; 4: mitochondria; 5: vacuole; 6: damaged organelle.

To further investigate the cellular effects of radiation on hydatid cysts, irradiated parasites and control were observed by thin section electron microscopy 24 hours after radiation. In control cysts, the germinal cells were intact, villi beneath the cuticle was visible. Organelles such as mitochondria and golgi with distinct outlines were distributed around the cell with ordered structure. In irradiated cysts, the germinal layer showed apparent damage. Abnormal appearance including sparse cytoplasms, absence of organelles such as mitochondria and golgi, and devoid of villi. These attributes are indicative of distressed or dying cells (Figure 3).

**Figure 4:**
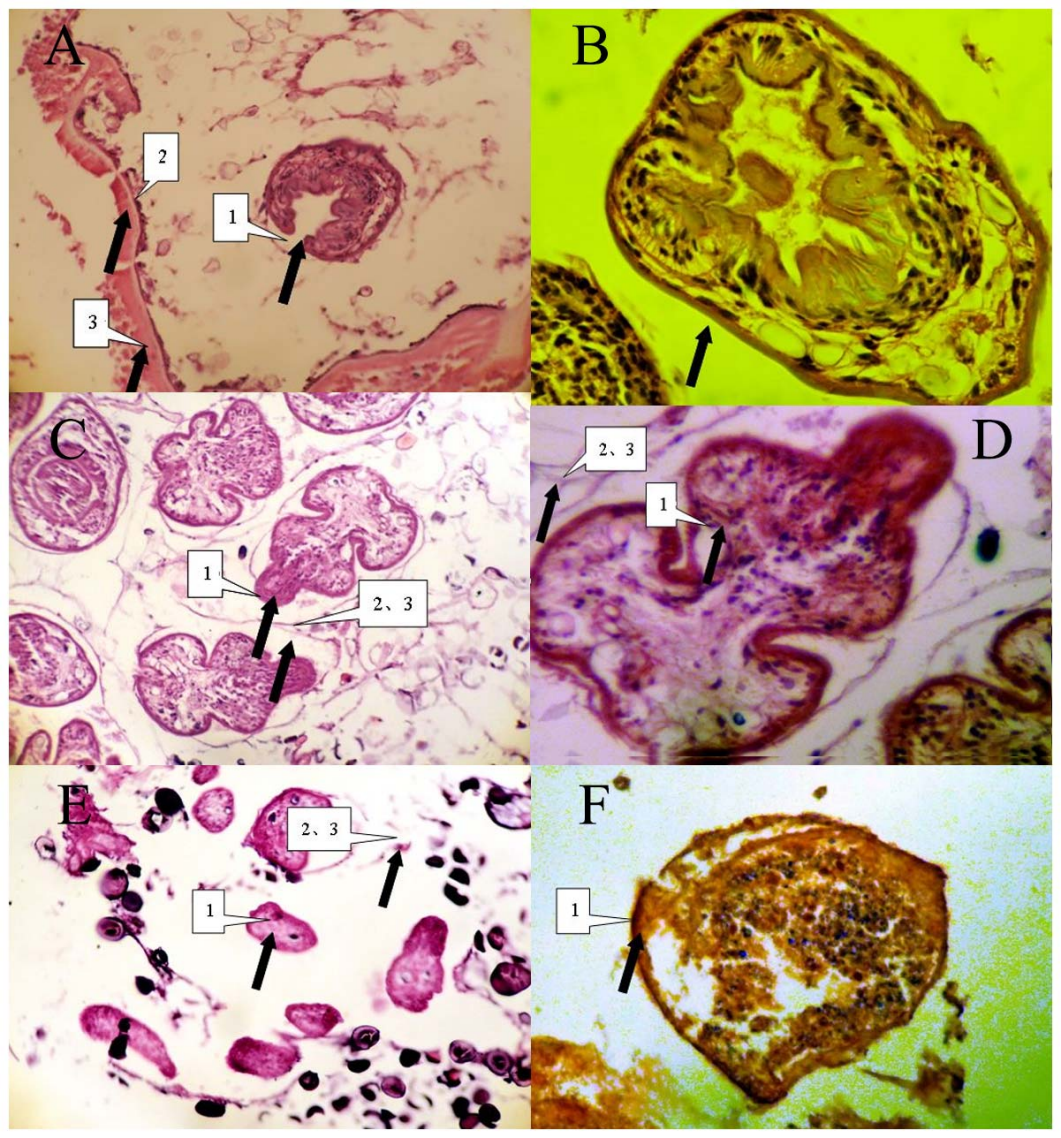
Representative light microscopy of *E. multilocularis* hydatid cysts from control and irradiated cultures. Examples of control (A and B), 30 Gy X-rays irradiated(C and D) and 30 Gy carbon-ion irradiated (E and F) parasites are shown. A, The protoscolex, germinal layer and cuticles were intact and clear (×40) in unirradiated samples. B. Protoscolex was visible and intact (×100). C and D, after 30 Gy X-rays irradiation, parasites displayed abnormal morphologies that included protoscolex eversion (1) and detachment of germinal layer from cuticles (2). E, After 30 Gy carbon-ion irradiation, loss of suction cups and scolex hooks, protoscolex contraction (3) were extensive in parasites.

The morphology was clear and intact under light microscope in control group. The protoscolex, germinal layer and cuticles were intact and clear. Abnormal protoscolex and detachment of germinal layer from cuticles were observed in X-rays irradiated group. Protoscolex contraction, loss of suction cups and scolex hooks were extensive in carbon-ion irradiated group (Figure 4).

### Radiation induced hydatid cysts apoptosis

**Figure 5:**
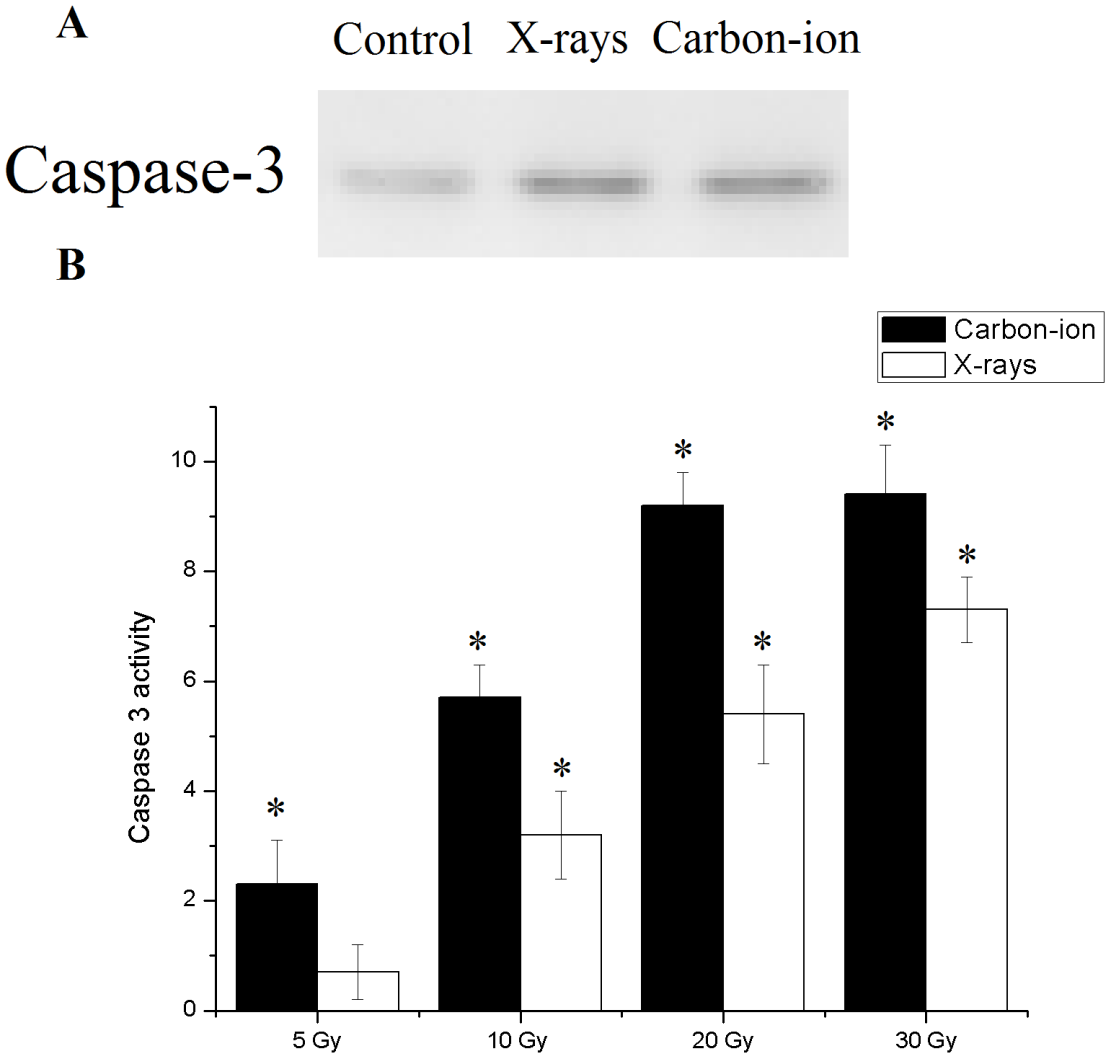
Apoptosis in 30 Gy X-rays and carbon-ion irradiated *E. multilocularis* hydatid cysts. A, Caspase 3 expression in hydatid cysts with and without 30 Gy ionizing radiation. B, Caspase 3 like activity was measured as the difference in *p*NA production between the samples with and without ionizing radiation. Error bars represent the SD, each done in at least triplicate. *Statistical significant at p<0.01.

Since the metabolic pathway of programmed cell is currently unknown in *E. multilocularis*, caspase 3, an effector molecule common to all know metabolic routs of apoptosis procedure was used as indicator of apoptosis. Caspase 3 expression was detected in hydatid cysts after 30 Gy carbon-ion or X-ray radiation, indicative of apoptotic index (Figure 5A).

The specific activity of active caspase 3 in irradiated cysts was higher than that in control. In addition, caspase 3 activity was more pronounced in carbon-ion irradiated cysts than X-rays irradiated cysts exposed to the same dose. Caspase 3 activity reached a plateau after 30 Gy radiation exposure (Figure 5B).

## Discussion

In this study, we provide the first comprehensive study on cellular and molecular alternations in *E. multilocularis* hydatid cysts treated with high doses of X-rays or carbon-ion radiation. Exposure of high doses radiation causes the attenuation of hydatid cysts. The morphological changes of hydatid cysts after radiation were similar to other reports using drugs [10], [11], confirming the inhibitory effect of radiation on hydatid cysts.

The significant decrease in mtDNA copy number indicates that the mtDNA degradation process becomes profound with increasing oxidative damage, presumably due to saturation of the repair capacity of mitochondria in *E. multilocularis* hydatid cysts. The persistent depletion in mtDNA content appears to be a direct consequence of active mtDNA degradation and may be the basis to the “persistent mtDNA damage” reported in several studies [12], [13]. The profound mtDNA damage and degradation then could lead to mitochondrial dysfunction and persistent oxidative stress [14]. Carbohydrates, as the main energy source of *E. multilocularis*, can be catabolized by arobotic respiration or two complementary anaerobic pathways. Mitochondrial dysfunction induced by radiation, thus could lead to severe hydatid cysts growth inhibition and cell death due to its extensive parasitic life style.

High level of apoptosis has been reported to be involved in hydatid cyst infertility in *Echinococcus granulosus* hydatid cysts [15], [16]. Lymphocytes apoptosis by modulator of hydatid fluid is reported to be one of the survival mechanisms for hydatid cysts [17]. These studies indicate that apoptosis play an important bifunctional role in hydatid cysts survival. Oxidative stress play a pivotal role in apoptosis induction [18]. Hanhua et al. reported that H_2_O_2_ and dexamethasone could induce the cellular apoptosis of protoscoleces [10]. However, oxidative stress induced apoptosis in *E. multilocularis* has not been reported. Here we found that ionizing radiation such as X-rays and carbon-ion irradiation could efficiently induce apoptosis in *E. multilocularis* hydatid cysts, which may caused by exacerbated oxidative stress.

Due to its physical and biologic advantages over conventional radiation therapy, heavy-ion therapy has high local control rates with relatively low toxicity compared with photo and proton radiation therapy [19], [20]. Our results also showed that carbon-ion radiation caused more severe damage on hydatid cysts than X-rays. However, the side-effect of heavy-ion therapy should not be ignored. Severe late complications has been reported in patients who received high dose heavy-ion radiation for esophageal cancer [20]. Further *in vivo* experimental data for the effects of heavy-ion therapy on hydatid cysts should be provided.

Our results provide a rationale future for exploring the application of radiotherapy as nonsurgical treatment method in treating this parasitic disease. Additionally, we find that carbon-ion radiation is more effective to damage hydatid cysts than X-rays, which may be a more suitable candidate for hydatid disease treatment.
